# Characterization of antibiotic resistance and host-microbiome interactions in the human upper respiratory tract during influenza infection

**DOI:** 10.1186/s40168-020-00803-2

**Published:** 2020-03-17

**Authors:** Lingdi Zhang, Christian V. Forst, Aubree Gordon, Gabrielle Gussin, Adam B. Geber, Porfirio J. Fernandez, Tao Ding, Lauren Lashua, Minghui Wang, Angel Balmaseda, Richard Bonneau, Bin Zhang, Elodie Ghedin

**Affiliations:** 1 Center for Genomics and Systems Biology, Department of Biology, New York University, New York, NY 10003 USA; 2 Department of Genetics and Genomic Sciences, Icahn Institute of Genomics and Multiscale Biology, Icahn School of Medicine at Mount Sinai, New York, NY 10029 USA; 3 Department of Epidemiology, School of Public Health, University of Michigan, Ann Arbor, MI 48109 USA; 4 National Virology Laboratory, Centro Nacional de Diagnóstico y Referencia, Ministry of Health, Managua, Nicaragua; 5 Sustainable Sciences Institute, Managua, Nicaragua; 6 Department of Epidemiology, School of Global Public Health, New York University, New York, NY 10003 USA

**Keywords:** Antibiotic resistance, Upper respiratory tract infection, Microbiome, Influenza infection, Metatranscriptome

## Abstract

**Background:**

The abundance and diversity of antibiotic resistance genes (ARGs) in the human respiratory microbiome remain poorly characterized. In the context of influenza virus infection, interactions between the virus, the host, and resident bacteria with pathogenic potential are known to complicate and worsen disease, resulting in coinfection and increased morbidity and mortality of infected individuals. When pathogenic bacteria acquire antibiotic resistance, they are more difficult to treat and of global health concern. Characterization of ARG expression in the upper respiratory tract could help better understand the role antibiotic resistance plays in the pathogenesis of influenza-associated bacterial secondary infection.

**Results:**

Thirty-seven individuals participating in the Household Influenza Transmission Study (HITS) in Managua, Nicaragua, were selected for this study. We performed metatranscriptomics and 16S rRNA gene sequencing analyses on nasal and throat swab samples, and host transcriptome profiling on blood samples. Individuals clustered into two groups based on their microbial gene expression profiles, with several microbial pathways enriched with genes differentially expressed between groups. We also analyzed antibiotic resistance gene expression and determined that approximately 25% of the sequence reads that corresponded to antibiotic resistance genes mapped to *Streptococcus pneumoniae* and *Staphylococcus aureus*. Following construction of an integrated network of ARG expression with host gene co-expression, we identified several host key regulators involved in the host response to influenza virus and bacterial infections, and host gene pathways associated with specific antibiotic resistance genes.

**Conclusions:**

This study indicates the host response to influenza infection could indirectly affect antibiotic resistance gene expression in the respiratory tract by impacting the microbial community structure and overall microbial gene expression. Interactions between the host systemic responses to influenza infection and antibiotic resistance gene expression highlight the importance of viral-bacterial co-infection in acute respiratory infections like influenza.

**Video abstract**

## Background

Influenza virus infection is commonly followed by secondary bacterial infection, leading to increased morbidity and mortality, particularly among susceptible groups including the elderly and the immunocompromised [1, 2]. Bacterial species with the potential to cause infections in the respiratory tract, such as *Haemophilus influenza*, *Staphylococcus aureus*, *and Streptococcus pneumonia*, are often found in association with influenza virus infections and have also been isolated from the upper respiratory tract of healthy individuals [3, 4]. The emergence and spread of antibiotic resistance strains of these potential pathogens, such as methicillin-resistant *Staphylococcus aureus* (MRSA) and multidrug resistance *Streptococcus pneumonia,* impact treatment and are of global concern.

Whereas the phenomenon of antibiotic resistance has been widely studied in the gut microbiome [5, 6], there have been limited attempts to profile antibiotic resistance gene (ARG) expression in the respiratory tract, the focus of the current study. The gut microbiome is considered to be a reservoir of antibiotic resistance, and studies have reported that antibiotics, by modifying the microbial community, can indirectly affect the immune response. This was demonstrated through the display of microbial-associated molecular patterns to the receptors on immune and epithelial cells in the host [7]. The possible consequence of this indirect response is varied stimulation of toll-like receptors, resulting in an altered immune response downstream [7]. Whether similar effects occur in the respiratory tract has not been fully addressed.

Since influenza virus infection impacts the host immune response and can lead to bacterial co-infection, we explored the interactions between the systemic host response to influenza virus infection and microbiome activities, including ARG expression in the airways, to better understand host-virus-bacteria dynamics during infection. We used novel data integration methods, such as Multiscale Embedded Gene Co-expression Network Analysis (MEGENA) [8], to build a host gene co-expression network that correlates host modules in influenza virus infection with microbial profiles, ARG expression, and microbial gene expression pathways.

## Results

### Microbial gene expression profiling clusters subjects into 2 groups

We analyzed samples from 37 subjects, including child index cases and their household contacts who developed influenza; 25 individuals were infected with influenza A H3N2 virus, 6 with influenza A H1N1pdm, and 6 with influenza B (Table S1). We obtained transcriptomic data of peripheral blood mononuclear cells (PBMCs) for 32 individuals, and metatranscriptomics and 16S rRNA gene amplicon profiles of respiratory samples for 35 individuals (Fig. S1).

**Fig. 1:**
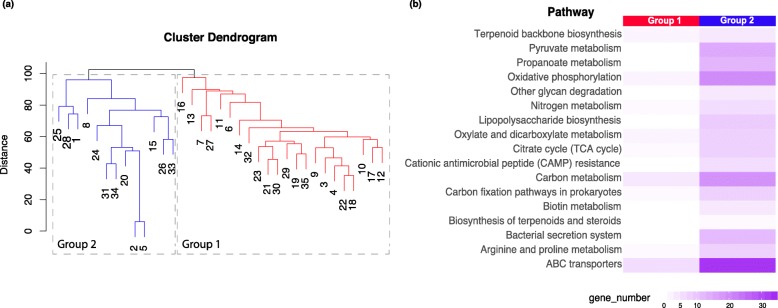
Microbial gene expression profiling of subjects. **a** The samples were clustered into two groups, with red indicating group 1 and blue indicating group 2. The groups were identified by using hierarchical clustering on the Euclidean distance between microbial gene expression profiles of the samples. **b** The pathways enriched with differentially expressed genes between group 1 and group 2 were plotted. The intensity of the color indicates number of genes being overexpressed in one group versus the other

We characterized overall microbial gene expression profiles by aligning metatranscriptomic sequence reads to the Kyoto Encyclopedia of Genes and Genomes (KEGG)-filtered Uniprot database from the Functional Mapping and Analysis Pipeline for metagenomics and metatranscriptomics studies (FMAP) [9]. Our samples clustered into 2 major groups (Fig. 1a) based on the hierarchical clustering of overall microbial gene expression profiles. We did pathway enrichment analysis on the genes differentially expressed between group 1 and group 2. Several bacterial pathways, including the ABC transporter pathway and the bacterial secretion system, were enriched with differentially expressed genes between the two groups (Fig. 1b).

**Fig. 2:**
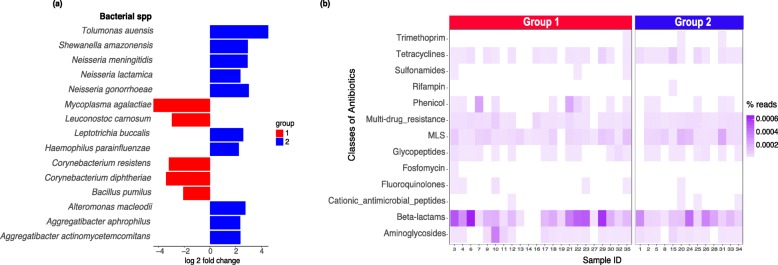
Associations between microbial gene expression, composition and antibiotic resistance. **a** Bacterial taxa with metatranscriptomic reads present at different relative abundance between the two groups were identified using DESeq2 and LEfSe, and plotted with log2 fold change. The red bars correspond to taxa for which genes were overexpressed in group 1 as compared to group 2, while the blue bars correspond to taxa overexpressed in group 2 compared to group 1. **b** Reads were assigned to antibiotic resistance genes (ARGs) by aligning to the MEGARes database; gene assignments were summarized at the level of classes of antibiotics to which the genes confer resistance. The heatmap shows the abundance of reads originating from antibiotic resistance genes relative to the total number of metatranscriptomic reads for each sample

By comparing the metatranscriptomic datasets between the two groups, we identified several bacterial genera, including *Corynebacterium*, *Neisseria*, *and Haemophilus*, that were differentially expressed. For example, *Corynebacterium* had a higher relative abundance of mapped RNA sequence reads in group 1, while *Neisseria* (primarily *N. meningitidis*) and *Haemophilus (H. parainfluenza*) had a higher relative abundance of mapped reads in group 2 (Fig. 2a).

To characterize antibiotic resistance gene expression profiles, we aligned the metatranscriptomic sequence reads (approximately 7.5 M reads per sample) to an antibiotic resistance database (MEGARes). On average, around 3100 reads per sample are aligned to the ARG database; assignment to an ARG required matches to at least two regions per gene, with a minimum of 10 mapped reads. Across the dataset, we identified genes that confer resistance to 13 classes of antibiotics (Fig. 2b) with variations across the samples. Genes identified that confer resistance to beta-lactam, aminoglycoside, tetracycline, and macrolide were also found to be expressed in another study that focused on the human gut microbiome [10]. Specific ARGs were also differentially expressed between the two groups identified by clustering the microbial gene expression profiles. For example, genes conferring resistance to phenicol were overexpressed in group 1 compared to group 2 (cutoff of 0.05 for Benjamini-Hochberg-corrected *p* values) (Fig. 2b).

### Commensal respiratory tract bacteria are correlated with antibiotic resistance gene expression

To study the correlations between ARG expression and bacterial taxa, we characterized the relative abundance of genera for each sample using the 16S rRNA gene profiles analyzed with the QIIME pipeline [11]. To determine whether any bacterial genera contributed to explaining specific variations in ARG expression, we used sparse partial least squares regression (spls) analysis [12], which can perform simultaneous dimension reduction and variable selection. We did a feature selection with spls using ARG expression profiles as response variables and the taxonomic assignments of operational taxonomic units (OTUs) from the 16S rRNA gene data as the predictors. Several bacterial genera correlated with specific ARG expression. For example, *Streptococcus* and *Moraxella* had positive correlations with the expression of beta-lactam resistance genes, while other common respiratory tract bacterial genera, such as *Neisseria,* had positive correlations with multidrug resistance gene expression (Table 1). Conversely, *Staphylococcus* had negative correlations with tetracycline resistance gene expression (Table 2). Metatranscriptomic reads that mapped to ARGs were further assigned to bacterial taxa using Kraken, a tool used to make taxonomic assignments from metagenomic type data [13]. A significant proportion of these reads mapped to *Streptococcus*, *Staphylococcus*, *and Neisseria.* On average, 47% of the reads originating from ARGs mapped to *Streptococcus*, while 10% of the reads mapped to *Staphylococcus* (Table S2). Thirty-four percent of the *Streptococcus* ARG reads mapped to *Streptococcus pneumonia*, while the majority of the *Staphylococcus* ARG reads corresponded to *Staphylococcus aureus* (Tables S3 and S4).

**Table 1:** Positive correlations between antibiotic resistance gene expression and bacterial taxa

Antibiotic resistance genes	^a^Taxa (positive associations)	*P* values	Correlation values
Beta-lactams	g_*Salinicoccus*	0.026	0.08
	f*_Gemellaceae*	0.030	0.05
	g_*Granulicatella*	0.034	0.04
	g_*Streptococcus*	< 0.0005	0.09
	g_*Methylobacterium*	0.024	0.05
	g_*Moraxella*	0.022	0.09
macrolide-lincosamide-streptogramin (MLS)	g_*Bifidobacterium*	0.038	0.05
	g_*Kaistobacter*	0.009	0.06
	g_*Sphingomonas*	0.022	0.07
	f_*Gemellaceae*	0.024	0.08
	*g_Bulleidia*	0.007	0.06
Tetracyclines	g_*Brachybacterium*	0.042	0.06
	g_*Tannerella*	0.006	0.06
	f_*Planococcaceae*	0.034	0.05
	f_*Aerococcaceae*	0.046	0.046
	g_*Selenomonas*	0.010	0.08
	g_*Leptotrichia*	0.033	0.08
	g_*Sphingomonas*	0.004	0.10
	g_*Deinococcus*	0.020	0.06
Multidrug resistance	g_*Rothia*	0.020	0.07
	f_*Gemellaceae*	0.009	0.08
	g_*Streptococcus*	0.045	0.06
	g_*Leptotrichia*	0.019	0.07
	g_*Neisseria*	0.035	0.09
	g_*Sphingomonas*	0.026	0.08
	g_*Erwinia*	0.036	0.09
	f_*Enterobacteriaceae*	0.041	0.10

^a^f_ and g_ indicate whether the taxonomic assignment was made at the family or genus level, respectively

**Table 2:** Negative correlations between antibiotic resistance gene expression and bacterial taxa

Antibiotic resistance genes	^a^Taxa (negative associations)	*P* values	Correlation values
Beta-lactams	g_*Staphylococcus*	0.047	− 0.04
	g_*Aerococcus*	< 0.0005	− 0.04
	f_*Rhodobacteraceae*	0.021	− 0.04
MLS	g_*Aeromicrobium*	0.030	− 0.13
	g_*Staphylococcus*	0.005	− 0.06
	f_*Peptostreptococcaceae*	0.045	− 0.11
Tetracyclines	g_*Kocuria*	0.049	− 0.07
	g_*Staphylococcus*	0.001	− 0.05
	g_*Mycoplasma*	0.001	− 0.10

^a^f_ and g_ indicate whether the taxonomic assignment was made at the family or genus level, respectively

### Host systemic responses to influenza infection are associated with microbial composition and gene expression

To investigate the correlations between the microbiome in the upper respiratory tract and systemic host responses stimulated by influenza virus infection, we analyzed host genes that were differentially expressed during influenza virus infection and after recovery. We also looked specifically at the correlations between host systemic responses and the respiratory tract microbiome and ARG expression. We analyzed patient blood samples collected 1–2 days after onset of symptoms and at 30–45 days—this last time point as a proxy of a pre-infection baseline—and determined global host transcriptome expression. We performed Multiscale Embedded Gene Co-expression Network Analysis (MEGENA) [8] on the host transcriptomic data, and correlated corresponding MEGENA modules with ARG expression, and taxonomic assignments from the 16S rRNA gene data.

We identified 567 co-expressed host gene modules (subnetworks); 38 of these were enriched with genes with a significant response to influenza virus infection (|FC| > = 2, 5% FDR) (Table S5). The samples were separated into group 1 and group 2 based on the metatranscriptomic data, and a differentially expressed gene (DEG) analysis from the host transcriptome was performed within each of the two groups. By overlapping the group-based DEGs with the DEGs in the 38 enriched host modules identified from all samples (Table S5), we identified some host modules with genes only upregulated or downregulated within one of the two groups (Table S6), such as modules M44, M45, and M46. This suggests a correlation between microbial gene expression and the host response to influenza virus infection.

**Fig. 3:**
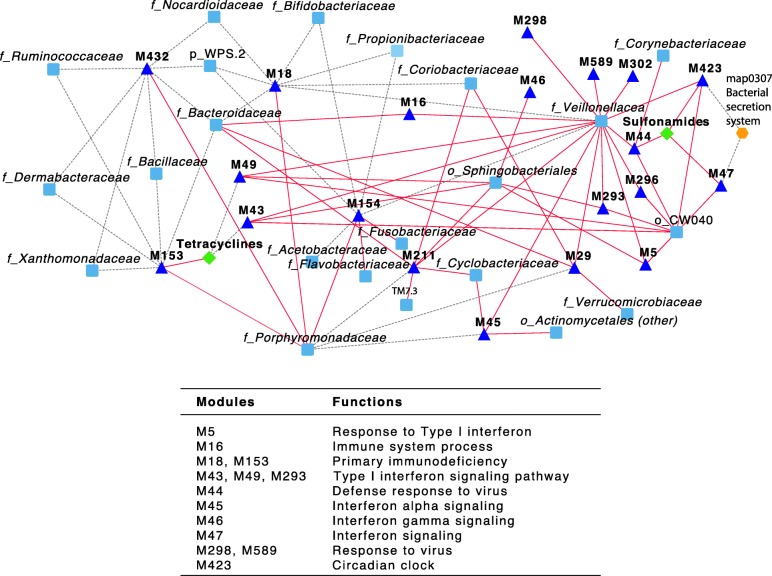
Network analysis of interactions between host gene expression, bacterial taxa, bacterial (metatranscriptome) gene expression, and antibiotic resistance. The modules indicated by blue triangles are co-expressed gene clusters in response to influenza virus infection. Direct and indirect interactions between the modules, antibiotic resistance (green diamonds), microbiome expressed functions (orange hexagons), and bacterial families (light blue squares) were identified by correlation analysis. The red edges indicate positive correlations and the grey dashed edges indicate negative correlations. The host modules and the pathways enriched with the genes in the modules are listed. Only the host modules with significantly enriched pathways are shown in the table. o_, p_, f_, and g_ indicate whether the taxonomic assignment was made at the order, phylum, family, or genus level, respectively

To further understand the interactions between the microbiome and the host response, we integrated data from bacterial taxa, bacterial pathways, and antibiotic resistance gene expression profiles with the top 20 host gene modules. We found significant correlations between host modules and bacterial families (Fig. 3). For example, *Veillonellaceae* had positive correlations with many host modules, including M49 and M5, which are enriched with genes involved in the type I interferon signaling pathway. We also observed correlations between specific host modules, bacterial pathways, and ARGs. For example, host module M47, also enriched with genes involved in interferon signaling, had a positive correlation with the expression of sulfonamide resistance genes and a negative correlation with the bacterial secretion system. Several key regulators of the network—i.e., hubs with high connectivity—were identified for each of the host modules. These key regulators are genes with the most impact on neighboring genes in the subnetworks. GBP1 (guanylate-binding protein 1), for example, appeared to be a key regulator in host modules M47 (Fig. S2) and M5, and was upregulated during influenza virus infection (FC = 3.5, adjusted *P* value = 0.003). GBP1 has a known function against influenza virus infection and it is associated with anti-viral and anti-bacterial functions. Thus, the correlations observed from the network between the host modules and the microbiome in the upper respiratory tract could be due to the direct impact of influenza virus infection on the host and the respiratory microbiome. The network topology suggests potential connections between specific host pathways, key regulators, the microbial community composition, and gene expression—including ARG expression—during influenza virus infection.

## Discussion

During influenza virus infection, interactions between the host, the virus, and resident bacteria with pathogenic potential are known to complicate and worsen disease. In this study, we identified associations between the microbiome of the upper respiratory tract, ARG expression, and host responses to influenza virus infection, providing some insight into potential interactions that contribute to disease severity.

We identified several genes as being differentially expressed between the two subject groups that were clustered based on their microbial gene expression profiles. Pathways found to be enriched included a number of genes involved in bacterial physiology, such as the bacterial secretion system, which is one of the strategies used by pathogenic bacteria to secrete virulence factors for host invasion [14, 15]. ABC transporters, which contribute to substrate transport across the bacterial membrane and are related to antibiotic resistance [16, 17], were also enriched with differentially expressed genes between the two groups. Several bacterial taxa were differentially abundant between the two groups, including *Staphylococcus*, *Pseudomonas*, and *Moraxella*, which have a number of species that can carry ARGs [18–21]. Incidentally, the microbial composition did not appear to be influenced by the type or subtype of influenza viruses that infected the individuals tested (data not shown).

Previous studies have reported on the dynamics of antibiotic resistance expression in the gut and in the environmental microbiome, and have shown the diversity in ARG expression and niche specificity [10, 22]. Other studies have shown the presence of ARGs in the lungs of cystic fibrosis patients [23], and in the stool of individuals who had never been exposed to antibiotics [24]. In our study, we identified bacteria-expressed genes that confer resistance to classes of antibiotics that the infected individuals were not taking, according to their antibiotic use history in the 12 months preceding influenza diagnosis. The three antibiotics reported to have been prescribed to this group of individuals include Beta-lactams, macrolides, and Nitroimidazole (Table S1), but ARGs were also found against classes of antibiotics that are currently rarely taken, such as phenicol. Although we may not have a complete record of past antibiotic use because antibiotics taken over-the-counter are often not reported, it is also possible that some of the ARGs expressed are unrelated to antibiotic use by the individuals in which these were detected and could be the result of transmission of antibiotic resistant strains between individuals. Transmission of drug-resistant bacteria both within the human population and between environmental reservoirs and humans is known to occur [25]. Some of the ARGs we identified in our samples are also simply efflux pumps that naturally function to extrude toxins and chemicals from the bacteria [26], and thus antibiotic resistance is a secondary effect.

We determined a number of correlations between antibiotic resistance and specific bacterial taxa. For example, *Moraxella* and *Streptococcus* had positive correlations with beta-lactam resistance gene expression. In several studies, species in these genera, such as *Moraxella catarrhalis* and *Streptococcus oralis*, were found to carry beta-lactam resistance genes [27–29]. Given that harboring ARGs can exact a fitness cost on the host bacteria [30], it is possible that other taxa compete with the bacteria that express or maintain the ARGs, leading to the observed negative associations. For example, we observed that the relative abundance of *Staphylococcus* and *Mycoplasma* determined from the 16S rRNA gene data was negatively correlated with tetracycline resistance gene expression. We also observed previously unreported correlations between ARG expression and several bacterial taxa, such as multidrug resistance and *Rothia*. Mapping of the ARG reads to the bacterial taxa databases also revealed an enrichment of *Streptococcus*, *Neisseria,* and *Staphylococcus,* especially clinically important bacterial species, such as *Streptococcus pneumonia* and *Staphylococcus aureus*. Indirect correlations suggest that although some taxa present in the respiratory tract do not specifically express ARGs, they could play an indirect role in ARG expression by affecting microbial composition and function.

Using gene co-expression network analysis, we characterized associations between host responses to influenza virus infection and the microbiome, as well as ARG expression in the upper respiratory tract. By integrating the microbiome information, we observed correlations between specific host modules and microbial community composition and gene expression. For example, *Veillonellaceae,* a family commonly represented in the respiratory tract, had positive correlations with some of the host modules that were enriched with genes involved in the response to viral and bacterial invasion, such as type I interferon signaling. Influenza virus is known to stimulate type I interferons and promote bacterial colonization in mice; type I interferon signaling is also known to have both protective and detrimental responses to bacterial infections depending on the invading pathogens [31]. The host module M47, enriched with genes involved in interferon signaling, also had strong correlations with ARGs against the sulfonamides and the bacterial secretion system. One of the key regulators in this host module, GBP1, was upregulated during influenza infection. GBP1 is a GTPase induced by interferon with antiviral and antimicrobial activities [32, 33] that can recruit NADPH enzyme components and antimicrobial peptides to help bacterial killing [34]. A recent study determined that even when there is no identifiable bacterial co-infection or super-infection following influenza virus infection, host responses to “bacterial” patterns are detected in whole blood transcriptomic analysis [35]. While the subjects in our study were not tested for bacterial infection, we observed correlations between host responses to influenza virus infection and specific bacterial taxa. Observations from the network analysis could be the result of how influenza virus infection impacts the host and the microbial community, and how, in return, the host alters the microbiome in the respiratory tract. The expression of some ARGs is regulated by signal transduction systems, such as the two-component systems, which are also involved in other physiological processes, such as adhesion and autolysis [36]. Correlations between the host response and ARG expression indicate an effect of the host on the microbiome, which in turn impacts ARG expression that can be regulated by the bacterial response to stress. Thus, the network suggests several specific connections between the host modules (especially the interferon signaling pathway and GBP1), the respiratory tract microbial composition, and the microbial gene expression.

The novel method we used to integrate information from the host responses to influenza infection with microbial composition and gene expression highlights the complex interplay between the microbial community and the influenza-infected host. However, there were a number of limitations to this study: First, the analysis was limited to sequence similarity to known ARGs, and thus novel antibiotic resistance genes could not be detected. It also did not include the analysis of point mutations that are known to be associated with antibiotic drug resistance, thus the profiling of ARG expression was conservative. Secondly, for the correlation analyses, we were unable to test for familial effects because of the limited number of index-household contact pairs. In a follow-up study that is currently ongoing, we collected respiratory samples from more than 50 subjects in 10 households, with multiple contact cases within households, including from healthy controls. This expanded study will also allow us to determine whether ARG transmission is a common occurrence and the respiratory tract an important reservoir of antibiotic resistance genes, as implicated by our current analysis.

## Conclusions

The analysis of individuals from a Nicaraguan household study reveals how host response to influenza virus infection could indirectly impact antibiotic resistance gene expression in the respiratory tract by affecting the microbiome. It highlights the importance of understanding these associations in individuals infected with influenza virus and possibly other respiratory viruses. The correlations identified between host responses to influenza infection and the bacteria present in the airways show the possible effects of the host responses on the microbiome. This is important as both the host response and the microbiome can influence the risk of secondary bacterial infections because of interactions between potentially pathogenic bacteria and the commensals present in the respiratory tract. This analysis also suggests that the respiratory tract is possibly an important reservoir of antibiotic resistance genes in humans and should be further explored, especially regarding inter-host transmission of antibiotic resistance genes during influenza epidemics.

## Materials and methods

### Sample collection

Samples were collected from 37 individuals participating in the Household Influenza Transmission Study (HITS) in Managua, Nicaragua. Respiratory specimens consisted of pooled nasal and throat swabs collected at 1–2 days post illness onset from individuals with influenza-like symptoms between July 2013 and October 2014 in Managua, Nicaragua. Samples were shipped to the Center for Genomics and Systems Biology, New York University, and stored at − 80 °C. The HITS sample cohort included child index cases enrolled in the Nicaraguan Influenza Cohort Study and their family members who developed influenza. An acute blood sample was collected from all participants at enrollment into the study, and a convalescent sample was collected 30–45 days later. Blood samples were immediately transferred to the Centro Nacional de Diagnostico y Referencia, the Nicaraguan National Laboratory, where peripheral blood mononuclear cells (PBMC) were separated using Leucosep tubes (Greiner Bio-One) containing 3 mL of Ficoll Histopaque (Sigma) following established protocols [37]. The PBMCs were stored in liquid nitrogen until shipment. The HITS study was approved by the institutional review boards at the Nicaraguan Ministry of Health and the University of Michigan. Informed consent or parental permission was obtained for all participants and children aged 6 years and older provided assent.

### RNA extraction and library preparation for metatranscriptome sequencing

Total RNA was isolated from 80% of the volume of each respiratory sample with the QIAGEN RNeasy Micro Kit (QIAGEN, Hilden, Germany) according to the manufacturer’s recommendations and stored at − 80 °C. No mRNA enrichment or rRNA depletion steps were performed due to the limited biomass of the starting material. Double-stranded cDNA was synthesized and amplified from 200–500 pg of input RNA with the NuGEN Ovation RNA-seq V2 kit (NuGEN Technologies, Inc., San Carlos, CA) and purified using the QIAquick PCR Purification Kit (QIAGEN, Hilden, Germany). cDNA was sheared to 250 bp using the S220 Focused-ultrasonicator (Covaris Inc., Woburn, MA) and purified using Agencourt RNAClean XP beads (Beckman Coulter, Inc., Brea, CA). Sequencing libraries were prepared from 100 ng of purified, sheared cDNA with the NuGen Ovation Ultralow System V2 1-16 (NuGEN Technologies, Inc., San Carlos, CA). Libraries were quantified by qPCR using the KAPA Library Quantification Kit (KAPA Biosystems, Wilmington, MA) on a Roche 480 LightCycler (Roche, Basel, Switzerland); their size distributions were measured on a 2200 TapeStation using a D1000 ScreenTape (Agilent Technologies, Santa Clara, CA). Libraries were diluted to 2 nM in dilution buffer (10 mM Tris, pH 8.5) and combined with equimolar input into 4 sequencing pools (9 libraries per pool). Paired-end sequencing (2 × 100 bp) was performed at the Genomics Core Facility (Center for Genomics and Systems Biology, New York University) on the Illumina HiSeq 2500 instrument with TruSeq SBS V3 chemistry according to the manufacturer’s instructions (Illumina, Inc., San Diego, CA). Each pool was run on one lane of a High Output flowcell.

### Host transcriptome library preparation and data processing

RNA was extracted from PBMCs with the RNeasy Mini Kit (QIAGEN, Hilden, Germany) according to the manufacturer’s recommendations and stored at − 80 °C. Sequencing libraries were prepared with the TruSeq RNA Library Prep Kit v2 (Illumina, San Diego, CA) according to the low sample (LS) protocol and with 1 μg input mass. Libraries were quantified by qPCR using the KAPA Library Quantification Kit (KAPA Biosystems, Wilmington, MA) on a Roche 480 LightCycler (Roche, Basel, Switzerland); their size distributions were measured on a Fragment Analyzer using the Standard Sensitivity NGS Fragment Analysis Kit (Advanced Analytical Technologies, Ankeny, IA). Libraries were diluted to 2 nM in dilution buffer and combined with equimolar input into 4 sequencing pools (14 libraries per pool). Paired-end sequencing (2 × 100 bp) was performed at the Genomics Core Facility (Icahn School of Medicine at Mount Sinai) on the Illumina HiSeq 2500 instrument with TruSeq SBS V3 chemistry according to the manufacturer’s instructions (Illumina, Inc., San Diego, CA). Each pool was run on one lane of a High Output flowcell. The raw sequencing reads were aligned to the human hg19 genome using star aligner (version 2.4.0 g1) guided by the Ensembl transcriptomic annotation model of GRCh37.70. After read alignment, featureCounts [38] was used to quantify expression at the gene level. Genes with at least 5 reads in at least 4 samples were considered expressed and hence retained for further analysis. The gene level read counts data was normalized using the trimmed mean of M-values normalization (TMM) method [39] to adjust for sequencing library size differences. Normalized read counts were further adjusted for batch effect using a linear model. The residuals from the regression model were used for downstream analysis. Differentially expressed genes were determined between acute and convalescent blood samples using linear models together with empirical Bayes statistics from the LIMMA R package [40]. DEGs were considered significant with a fold change (FC) cutoff of |FC| ≥ 2 and an FDR-corrected *p* value of 0.05 or less.

### DNA extraction and 16S rRNA gene sequencing

Genomic DNA was isolated from the remaining volume of each sample with the PowerSoil DNA Isolation Kit (Qiagen) and stored at − 20 °C. The 16S rRNA gene V4 region was amplified according to previously published methods [41] using Q5 Hot Start High-Fidelity DNA Polymerase (New England BioLabs Inc., Ipswich, MA). Reactions were purified using 0.65× volumes of Agencourt RNAClean XP beads (Beckman Coulter, Inc., Brea, CA). Each sample was quantified using the Qubit 2.0 Fluorometer (ThermoFisher Scientific, Inc., Waltham, MA), pooled with equal input mass and repurified with 0.65× volumes of AMPure XP beads (Beckman Coulter, Inc., Brea, CA). The final sequencing pool was quantified by qPCR with the KAPA Library Quantification Kit (KAPA Biosystems, Wilmington, MA) on a Roche 480 LightCycler (Roche, Basel, Switzerland). The library was sequenced at the Genomics Core Facility (Center for Genomics and Systems Biology, New York University) using an Illumina PE 2 × 250 V2 kit on an Illumina MiSeq Sequencer (Illumina, Inc., San Diego, CA).

### Metatranscriptome gene expression profiling and antibiotic resistance gene identification

The metatranscriptomic sequencing reads were demultiplexed, and quality was assessed using the FastQC Toolkit (http://www.bioinformatics.babraham.ac.uk/projects/fastqc/). Contaminating human, ribosomal RNA, and adaptor sequences were removed from each dataset using DeconSeq [42], SortMeRNA [43], and Trimmomatic [44], respectively. The duplicated reads were removed by using Fastuniq [45]. The filtered reads were assigned to the orthologous gene families using the FMAP bioinformatics tool (FMAP: Functional Mapping and Analysis Pipeline for metagenomics and metatranscriptomics studies) and with the default KEGG-filtered Uniref90 reference cluster [9]. The reads were mapped to the database using the DIAMOND [46] mapping program with default parameters. The KEGG orthologous gene family abundance file for each sample was generated using the FMAP_quantification.pl script with default parameters. The pathways enriched with differentially expressed microbial genes between the two groups were also identified using the FMAP_comparison.pl and FMAP_pathway.pl scripts with 5% FDR.

The antibiotic resistance genes were annotated by aligning the filtered metatranscriptomic reads to the antibiotic resistance gene database, MEGARes [47]. The reads assigned to genes that confer resistance only by mutation were removed from the final results (as these genes are unrelated to antibiotic resistance when they do not carry mutations). The alignment was done on the Burrows-Wheeler Aligner (BWA) [48] using the BWA-MEM alignment method and default parameters. The alignments were filtered such that each antibiotic resistance gene needed to have matching reads in at least two regions for the reads to be kept, and the antibiotic resistance gene matrix was filtered so that each gene needed to have at least ten matching reads.

### 16S rRNA gene taxonomy assignments and multidimensional scaling (MDS)

The 16S rRNA gene sequencing data was processed through the Quantitative Insights into Microbial Ecology (QIIME) [11] pipeline. The reads were joined by using join_paired_ends.py and demultiplexed with split_library_fastq.py, and the chimeric sequences were identified and filtered out with ChimeraSlayer [49]. The sequences were clustered into operational taxonomic units (OTUs) at a 97% similarity threshold with the closed reference OTU picking method and the greengene database (gg_13_8_otus) [50]. A negative control with water replacing DNA was used to detect potential contamination from the reagents; however, no bacterial sequence passed the quality filter in the pipeline. Taxonomic assignment of the representative sequence from each OTU was made using the Ribosomal Database Project (RDP) classifier [51]. Taxonomic information was summarized at the genus and family levels using summarize_taxa.py. The biom file output from QIIME was analyzed in Rstudio [52] using the phyloseq [53] R package. Bray-Curtis dissimilarities were calculated, and the MDS plot was generated, based on the distance matrix on the samples (data not shown).

### Sample clustering by gene expression profiles and statistical analysis

The samples were clustered by using the gene expression profiles with hierarchical clustering on the Euclidean distance. DESeq2 [54] was used to identify differentially expressed ARG genes between the groups. The FDR-corrected *p* value of 0.05, combined with a log2 fold change greater than 2 or smaller than − 2, was used as thresholds to filter the significantly differentially expressed genes. The bacterial taxa with differential abundant metatranscriptomic reads between the two groups were identified by overlapping the results from DESeq2 and linear discriminant analysis effect size (LEfSe) [55].

### Taxonomy assignments on reads classified as antibiotic resistance genes and the metatranscriptomic datasets

The metatranscriptomic reads classified as antibiotic resistance genes were extracted from the datasets; taxonomic assignments were made to these reads using Kraken [13] with default parameters. The percentage of reads assigned to each taxon was calculated. Taxonomy assignments on all the metatranscriptomic reads in the datasets were also made by using Kraken.

### Associations between antibiotic resistance gene expression and bacterial genera

The genus-level assignments from the 16S rRNA gene profiles were centered log-ratio (clr) transformed, and filtered to the genera, which are highly variable. The analysis was performed by combining the compPLS [56] and spls packages in Rstudio [52] with the antibiotic resistance gene expression profiles as the response matrix, and the filtered 16S rRNA gene profiles as predictors.

Feature selection in spls was done using 10-fold cross validation to tune the parameters (sparsity); the latent component number was decided by decomposing the covariance. Bootstrapping and random permutation were used to determine the significance of a feature’s contribution to the model. The bacterial genera with bootstrapping *p* values smaller than 0.05 were considered statistically significant. As only genes conferring resistance to beta-lactam, tetracyclines, MLS, and multidrug resistance are abundant enough to yield good predictions, these four classes of antibiotics were kept with the identified associations.

### Gene co-expression network analysis

Multiscale Embedded Gene Co-Expression Network Analysis (MEGENA) [8] was performed to identify host modules of highly co-expressed genes in influenza infection. The MEGENA workflow comprises 4 major steps: (1) Fast Planar Filtered Network construction (FPFNC), (2) Multiscale Clustering Analysis (MCA), (3) Multiscale Hub Analysis (MHA), and (4) Cluster-Trait Association Analysis (CTA). The total relevance of each consensus module to influenza infection was calculated by summarizing the combined enrichment of the differentially expressed gene (DEG) signatures and trait correlations: ${G}_j=\prod \limits_i{g}_{ji}$, where *g*_*ji*_ is the relevance of a consensus ***j*** to a signature ***i;****g*_*ji*_ is defined as $\left({\max}_j\left({r}_{ji}\right)+1-{r}_{ji}\right)/\sum \limits_j{r}_{ji}$, where *r*_*ji*_ is the ranking order of the significance level of the overlap between the consensus module ***j*** and the signature.

### Correlation between gene co-expression network modules and traits

Correlations between modules and traits (ARG expression, bacterial taxa abundance, and metatranscriptome expression) were calculated as follows: Spearman’s correlation between the first principal component of the module gene expression and the corresponding traits was determined. In the case of metatranscriptome expression, KEGG reactions were considered as traits. In a second step, KEGG reactions that were significantly correlated with a particular module were then further tested for enrichment with KEGG pathways. All module/trait correlation *p* values were corrected using perturbation of the input data.

### Identification of enriched DEGs, pathways, key regulators in the host modules, and bacterial genera associated with host modules

To functionally annotate gene signatures and gene modules identified in this study, enrichment analysis using Fisher’s exact test (FET) was performed of the established pathways and signatures—including the gene ontology (GO) categories and MSMicrobial gene expression profiling of subjectsigDB—and the subject area-specific gene sets—including influenza host factors, Inflammasome, Interferome, and InnateDB. The hub genes in each subnetwork were identified using the adopted Fisher’s inverse chi-squared approach in MEGENA; Bonferroni-corrected *p* values smaller than 0.05 were set as the threshold to identify significant hubs.

## Supplementary information

**Additional file 1: Table S1.** Metadata for the participants involved in this study. Study IDs have been reassigned to the samples for simplicity. All the figures show only the study IDs.

**Additional file 2: Table S2.** Taxonomic assignments of ARG reads. The taxa were ranked based on the percentage of reads assigned from highest to lowest.

**Additional file 3: Table S3.** Percentage of ARG reads assigned to *Staphylococcus*. The species were ranked based on the percentage from highest to lowest.

**Additional file 4: Table S4.** Percentage of ARG reads assigned to *Streptococcus*. The species were ranked based on the percentage from highest to lowest.

**Additional file 5: Table S5.** DEGs in the host modules enriched with differentially expressed genes. Pathway enrichment analysis was done on the genes in the host modules with significant responses to influenza infection. Corrected p-values of 0.05 were used as cut off and the pathways with non-significant p-values are shown as N/A.

**Additional file 6: Table S6.** DEGs in the host modules upregulated and downregulated within the groups identified from metatranscriptomic data. Host modules with significant responses to influenza infection (Table S5) were further subjected to enrichment analysis for up- and down-regulated and group specific DEGs. Empty fields indicate non-significant enrichment. A cutoff of 0.05 for corrected p-values was used after FET.

**Additional file 7: Figure S1.** Diagram of the analysis pipeline for this study. Transcriptomic data of peripheral blood mononuclear cells (PBMCs), metatranscriptomic and 16S rRNA gene amplicon profiles of respiratory samples were generated. The analysis pipeline and data integration schema for these different types of data are shown.

**Additional file 8: Figure S2.** Subnetworks of genes clustered in M47. Each node in the graph represents one gene and it is labeled with the gene name. Red nodes are up-regulated genes and blue nodes are down-regulated genes comparing samples collected 1-2 days after onset of symptoms and samples collected at 30-45 days. The diamond shaped nodes are key regulators while the circular nodes are regular genes in the network. The sizes of the nodes represent node degree.

## Data Availability

The sequencing data in this study are available in the Sequence Read Archive (SRA). Metatranscriptome data and the 16S rRNA gene data are under accession numbers GSE126901 and GSE126900 (BioProject PRJNA523620), and the mRNAseq data under accession number GSE114588 (BioProject PRJNA471855). Scripts generating the data are available on GitHub (https://github.com/GhedinLab/Nicaragua_microbiome_flu_analysis).

## Abbreviations

ARG: Antibiotic Resistance Gene
BWA: Burrows-Wheeler Aligner
CTA: Cluster-Trait Association Analysis
DEG: Differentially expressed genes
FPFNC: Fast Planar Filtered Network Construction
FET: Fischer’s exact test
FMAP: Functional Mapping and Analysis Pipeline
GO: Gene Ontology
KEGG: Kyoto Encyclopedia of Genes and Genomes
LEfSe: Linear discriminant analysis effect size
MCA: Multiscale Clustering Analysis
MDS: Multidimensional scaling
MHA: Multiscale Hub Analysis
MEGENA: Multiscale Embedded Gene Co-Expression Network Analysis
MLS: Macrolide-lincosamide-streptogramin
OTU: Operational taxonomic unit
PBMC: Peripheral blood mononuclear cells
QIIME: Quantitative Insights Into Microbial Ecology
RDP: Ribosomal Database Project
spls: Sparse partial least squares regression
