# Supplementary figures and images for: Characterization of antibiotic resistance and host-microbiome interactions in the human upper respiratory tract during influenza infection
Source: Microbiome. 2020 Mar 17;8:39. doi: 10.1186/s40168-020-00803-2 (PMC7076942; doi:10.1186/s40168-020-00803-2)

Fig. S1

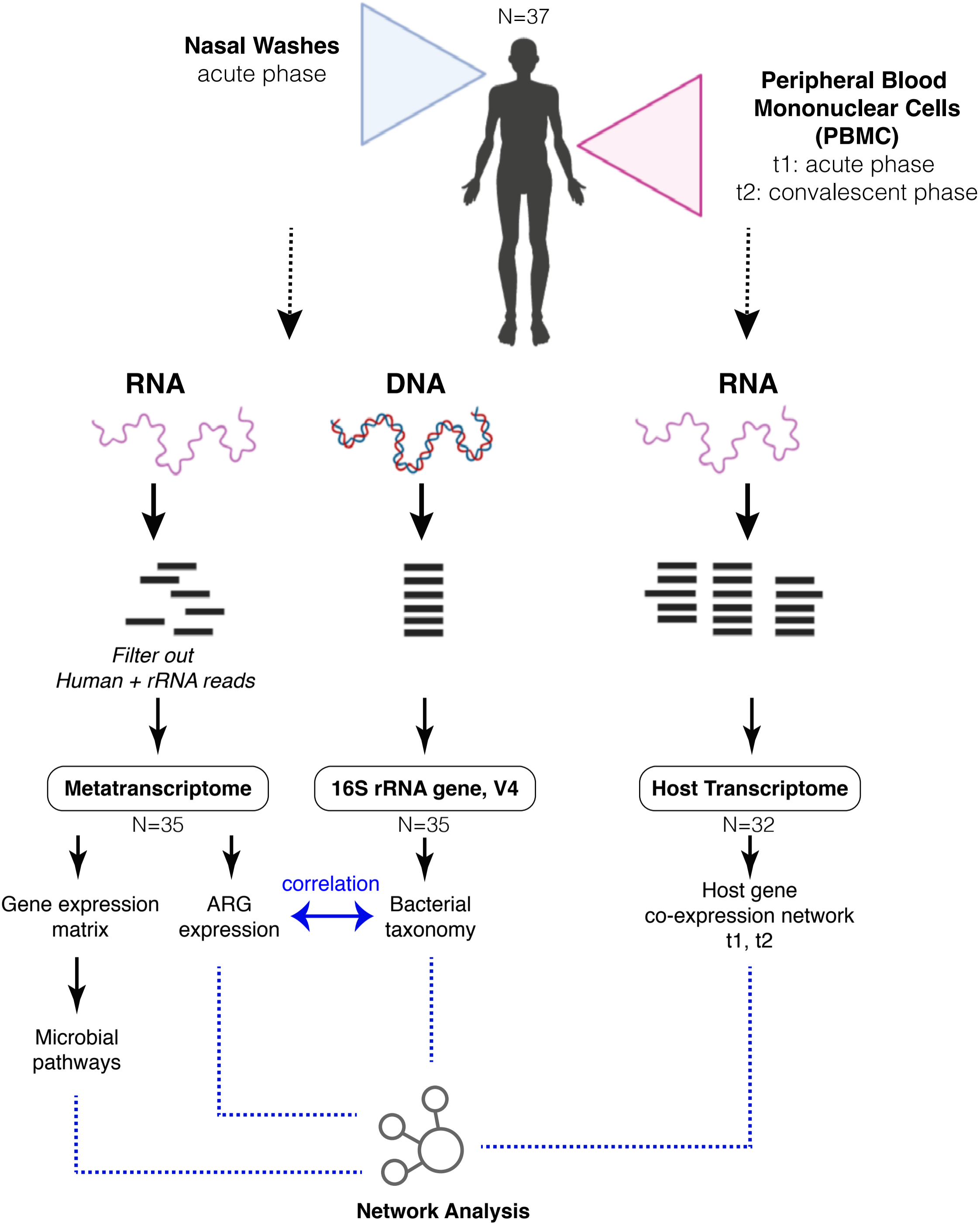

Supplement: Supplementary file 8 — Additional file 7: Figure S1. Diagram of the analysis pipeline for this study. Transcriptomic data of peripheral blood mononuclear cells (PBMCs), metatranscriptomic and 16S rRNA gene amplicon profiles of respiratory samples were generated. The analysis pipeline and data integration schema for these different types of data are shown. [file 40168_2020_803_MOESM7_ESM.pdf]

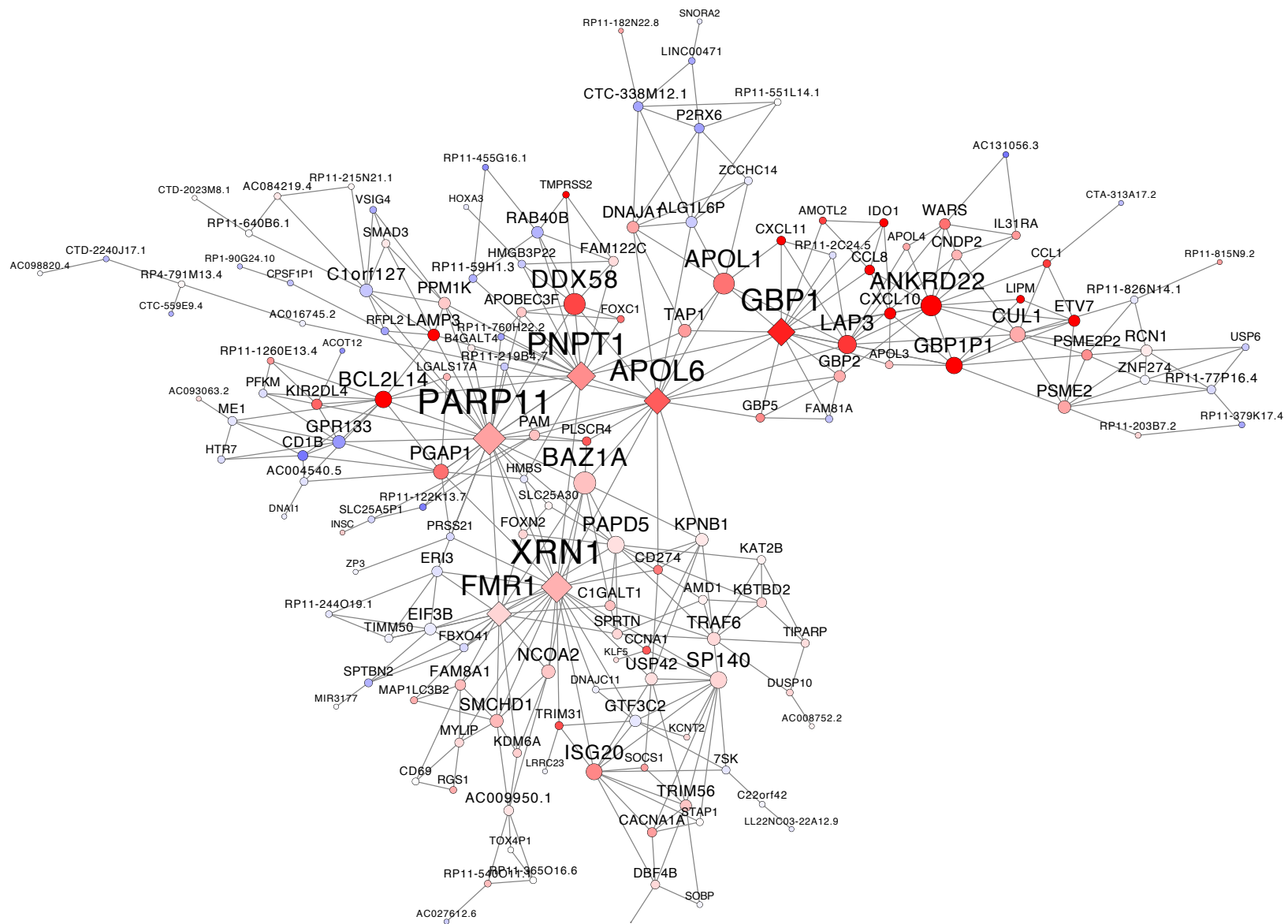

Supplement: Supplementary file 9 — Additional file 8: Figure S2. Subnetworks of genes clustered in M47. Each node in the graph represents one gene and it is labeled with the gene name. Red nodes are up-regulated genes and blue nodes are down-regulated genes comparing samples collected 1-2 days after onset of symptoms and samples collected at 30-45 days. The diamond shaped nodes are key regulators while the circular nodes are regular genes in the network. The sizes of the nodes represent node degree. [file 40168_2020_803_MOESM8_ESM.pdf]
